# Measuring Dementia Care Partners’ Fall Risk Management Behaviors: Development and Content Validation

**DOI:** 10.1093/geroni/igaf122.1570

**Published:** 2025-12-31

**Authors:** Yuanjin Zhou, Xiaoyi Zeng, Amanda Kilcrease, Noah Meah, Seung Choi, Elizabeth Phelan

**Affiliations:** The University of Texas at Austin, Austin, Texas, United States; The University of Texas at Austin, Austin, Texas, United States; The University of Texas at Austin, Austin, Texas, United States; The University of Texas at Austin, Austin, Texas, United States; The University of Texas at Austin, Austin, Texas, United States; University of Washington, School of Medicine, Seattle, Washington, United States

## Abstract

Family/friend care partners play a critical role in fall risk management (FRM) for older people with dementia via adopting FRM behaviors. No measure exists to assess these behaviors, hindering the development of effective strategies for engaging dementia care partners in FRM. The study aimed to develop a Fall Risk Management Scale (FRMS) for dementia care partners and improve its content validity. Based on our prior formative research, we drafted the 46-item FRMS that consists of eight sub-scales that assess multiple domains of FRM behaviors. To enhance the content validity, we performed two main activities: (1) we conducted a self-administered, written survey with 22 healthcare professionals to rate each item’s clarity, relevance, and importance using a four-point ordinal scale, followed by interviews to gather additional feedback; and (2) we carried out two rounds of cognitive interviews with 21 dementia care partners to explore their understanding of each item and overall scale. The Item-Content Validity Indices (I-CVI) from the survey, along with the issues identified in the interviews using the Question Appraisal System, guided the iterative revision of the scale. Although only 19 items had I-CVIs lower than 0.78, all items showed at least one problem that required revision. Commonly identified problems included unclear/vague wording and inappropriate assumptions. Care partners generally understood items as intended but requested adding examples to ensure their understanding. Over the two-round process, we added eight items, removed one, and revised all items. Our study improved the FRMS’s content validity for the next steps in its psychometric evaluation.

